# Amyloid and Tau Positron Emission Tomography Imaging in Alzheimer’s Disease and Other Tauopathies

**DOI:** 10.3389/fnagi.2022.838034

**Published:** 2022-04-22

**Authors:** Cinzia Maschio, Ruiqing Ni

**Affiliations:** ^1^Institute for Regenerative Medicine, University of Zurich, Zurich, Switzerland; ^2^Institute for Biomedical Engineering, ETH Zürich and University of Zurich, Zurich, Switzerland

**Keywords:** Amyloid-beta, tau, Alzheimer’s disease, biomarker, positron emission tomography, binding sites, affinities

## Abstract

The detection and staging of Alzheimer’s disease (AD) using non-invasive imaging biomarkers is of substantial clinical importance. Positron emission tomography (PET) provides readouts to uncover molecular alterations in the brains of AD patients with high sensitivity and specificity. A variety of amyloid-β (Aβ) and tau PET tracers are already available for the clinical diagnosis of AD, but there is still a lack of imaging biomarkers with high affinity and selectivity for tau inclusions in primary tauopathies, such as progressive supranuclear palsy (PSP), corticobasal degeneration (CBD) and Pick’s disease (PiD). This review aims to provide an overview of the existing Aβ and tau PET imaging biomarkers and their binding properties from *in silico*, *in vitro*, and *in vivo* assessment. Imaging biomarkers for pathologic proteins are vital for clinical diagnosis, disease staging and monitoring of the potential therapeutic approaches of AD. Off-target binding of radiolabeled tracers to white matter or other neural structures is one confounding factor when interpreting images. To improve binding properties such as binding affinity and to eliminate off-target binding, second generation of tau PET tracers have been developed. To conclude, we further provide an outlook for imaging tauopathies and other pathological features of AD and primary tauopathies.

## Introduction

Neurodegenerative diseases such as Alzheimer’s disease (AD) are associated with cognitive impairment and have a prevalence of 60 – 80% in individuals over 65 years of age. AD is the most common cause of dementia (Scheltens et al., 2021). Apart from the challenges imposed on people with AD and their relatives, the disease also places a significant financial burden on societies. The AD continuum consists of three phases: the preclinical, mild cognitive impairment (MCI) and dementia stages, where the last stage is further subdivided into mild, moderate and severe AD (Scheltens et al., 2021). The molecular hallmarks of AD are extracellular Aβ plaques and neurofibrillary tangles (NFTs) formed by hyperphosphorylated tau (Bloom, 2014). Although no impairment of memory manifests in the preclinical stage, pathological changes such as abnormal accumulation of Aβ and tau in the brain have already occurred, which necessitates early diagnosis and adequate interventions (Dubois et al., 2021). Frontotemporal dementia (FTD) includes a spectrum of primary tauopathy diseases featured by progressive executive, behavioral and language dysfunction, including progressive supranuclear palsy (PSP), corticobasal degeneration (CBD), and Pick’s disease (PiD) (Boeve et al., 2022).

Neuroimaging techniques enable us to non-invasively reveal information about the anatomy, metabolism, or activity of the brain. Multimodal imaging methods have emerged to (differentially) diagnose AD. Among the most commonly used imaging methods in the early diagnosis of AD are Aβ-positron emission tomography (PET), tau PET, FDG-PET, and volumetric magnetic resonance imaging (MRI) (Chételat et al., 2020). T_1_-weighted MR imaging enable detection of regional atrophy indicative of neurodegeneration, such as in the medial temporal lobe typical in AD. The use of various imaging modalities evidently focuses on detecting aberrant features of AD and gathering further knowledge on how protein aggregates associate with pathophysiology. [^18^F]fluoro-2-deoxy-D-glucose (FDG) PET has been applied for assisting the diagnosis of AD for many years by identifying cerebral hypoglucose metabolism. In addition to the aforementioned imaging biomarker, cerebrospinal fluid (CSF) Aβ (Aβ_42_/Aβ_40_ ratio, Aβ_42_) and phosphorylated tau (P-tau) biomarkers have been established in the routine clinical practice for assisting the diagnosis of AD (Hansson et al., 2019, 2021). The amount of soluble tau in the CSF serves as an early biomarker in AD patients as the level of phosphorylated and non-phosphorylated tau is associated with the progression of cognitive decline. Plasma biomarkers such as plasma p-tau (p-tau181, p-tau217) have emerged as promising biomarker ideal for large scale screening (Zetterberg and Blennow, 2020). As increasing levels of p-tau in blood correlate with the stage of the disease, p-tau might be a useful biomarker to assess disease progression (Palmqvist et al., 2021). In 2011, the National Institute on Aging and Alzheimer’s Association (NIA-AA) established a framework that consists of recommendations for identifying preclinical stages of AD with biomarkers (Jack et al., 2018). *In vivo* PET imaging has been included in the diagnostic criteria of neurodegenerative disorders to increase the accuracy and facilitate differential diagnosis (Hampel et al., 2021). The AT(N) framework, implemented in 2018, uses biomarkers rather than clinical symptoms to diagnose AD in living people by detecting Aβ aggregates as pathological biomarker, tau and neurodegeneration (Jack et al., 2018). Its purpose is to serve general clinical practice and provide a tool to diagnose and stage AD *in vivo*. In the continuum of AD, quantitative measures help in identifying the severity and stage of disease development. Evolved from the AT(N) framework, the ATX(N) system aims to introduce other pathological pathways, such as neuroinflammation, neurovascular dysfunction and blood–brain barrier (BBB) dysfunction, which in turn allows for the development of new biomarkers (Hampel et al., 2021). Recently, new synaptic vesicle glycoprotein 2A (SV2A) tracers such as [^11^C]UCB-J have been introduced, which has the potential as a biomarker for the decreased synaptic density in AD (Naganawa et al., 2021; O’Dell et al., 2021). In addition, PET tracers for microgliosis and astrocytosis have been increasingly utilized to provide tools for studying the pathophysiological events in AD (Rodriguez-Vieitez et al., 2016; Leng and Edison, 2021). Here, we focus on the Aβ and tau imaging tracers in regards to their *in vitro* and *in vivo* binding properties.

## Aβ Positron Emission Tomography Imaging

Amyloid precursor protein (APP) is a transmembrane protein expressed in neurons and other tissues, and once cleaved by γ- and β-secretases, it leads to the formation of a 37–49 amino acid long Aβ peptide (Mucke and Selkoe, 2012). Aβ can self-assemble into oligomeric Aβ with less well-defined structures and greater toxicity to cells as well as into fibrillar Aβ (Cohen et al., 2013). Different forms of Aβ plaques, including core plaque, diffuse plaque, and cerebral amyloid angiopathy (CAA) were observed in the autopsy brain (Thal et al., 2002).

### *In silico* and *in vitro*

Aβ imaging tracers detect β-sheets of Aβ fibrils and are mainly derivatives of benzoxazole and benzothiazole, and (Okamura et al., 2004). Three tracers, [^18^F]flutemetamol (Vizamyl), [^18^F]florbetaben (AV-1, Neuraceq), and [^18^F]florbetapir (Amyvid) have been approved by the Food and Drug Administration (FDA) and European Medicines Agency (EMA) for clinical use. [^11^C]PiB, a thioflavin T derivative, is the most commonly used tracer for *in vivo* Aβ PET imaging in a research setting. Klunk et al. (2004) reported increased [^11^C]PiB abundance in the cortical regions of AD patients compared to non-demented controls. [^11^C]PiB preferentially binds to Aβ_42_ fibrils with one high and one low binding site (Fodero-Tavoletti et al., 2007; Ni et al., 2013, Ni et al., 2017). Apart from labeling classical plaques, [^11^C]PiB also detects diffuse plaque and, to a minor extent, NFT. In addition, [^11^C]PiB detects CAA, a comorbidity that accumulates mainly in the occipital lobe (Johnson et al., 2007). Recent study showed that [^11^C]PiB PET can underestimate brain Aβ burden in the presence of cotton wool plaques (Abrahamson et al., 2021). [^18^F]AZD4694, a benzofuran derivative, structurally resembles [^11^C]PiB (Rowe et al., 2013), enabling the detection of small Aβ aggregates and minimally recognizing white matter. Examination of the regional distribution in the brains of AD patients compared to healthy controls indicated similar binding properties between [^18^F]AZD4694 and other Aβ radioligands, such as [^11^C]PiB (Cselényi et al., 2012). Binding characterization study showed that [^3^H]florbetaben bound to Aβ deposits in AD brain with high affinity and was able to accurately detect the Aβ burden in AD and shows low non-specific binding to non-demented control (Ni et al., 2021). [^18^F]florbetapir, a stilbene derivative, demonstrated high-affinity binding to Aβ, and faster uptake in the brain than [^18^F]florbetaben and [^18^F]flutemetamol (Mason et al., 2013). The radioligand [^11^C]BF227, a benzoxazole derivative, showed acceptable BBB permeability and Aβ affinity and preferentially bound to dense plaques in AD patients (Kudo et al., 2007).

### In vivo

High concordance between Aβ PET tracers have been reported. A study comparing [^11^C]PiB and [^18^F]flutemetamol in MCI, AD and healthy subjects exhibited promising outcomes regarding [^18^F]flutemetamol’s specificity (85.3%) and sensitivity (97.2%) in differentiating AD from controls (Hatashita et al., 2014). High concordance between [^11^C]PiB and [^18^F]flutemetamol uptake was reported (0.81) in this study (Hatashita et al., 2014). Similar robust concordance in the tracer brain uptakes have been reported in head-to-head studies using different pairs of amyloid tracers, e.g., [^11^C]PiB vs. [^18^F]florbetapir (Su et al., 2019), and [^18^F]flutemetamol vs. [^18^F]florbetaben (Cho et al., 2020). In regards to the brain uptake of the tracers, [^18^F]florbetapir showed a lower cortical uptake (standardized uptake value, SUV = 1.67) compared to [^18^F]flutemetamol (SUV > 2) (Choi et al., 2009). The off-target binding of [^18^F]-labeled tracers to white matter is a common but unwanted limitation in regard to PET imaging tracers. To facilitate the discrimination between Aβ positivity or negativity scans and the standardization of rating methods, a quantitative threshold to assess Aβ abundance is needed. Therriault et al. (2021) suggested a standard uptake value ratio (SUVR) of 1.55 for Aβ positivity for [^18^F]AZD4694, SUVRs of 1.1 for low Aβ burden, and 1.24 for established pathology for [^18^F]florbetaben (Bullich et al., 2021). Similarly, the centiloid method suggested the use of the unit to quantitatively determine the Aβ load of PET images using a standardized approach. The scale ranges from 0 to 100, where 0 anchors reflect amyloid-negative outcomes, and 100 anchors reflect typical amyloid-positive AD patients. Standardization of acquisition time duration, tracer usage and reconstruction algorithms facilitate multicentric collaborations (Klunk et al., 2015).

## Tau Positron Emission Tomography Imaging

**FIGURE 1:**
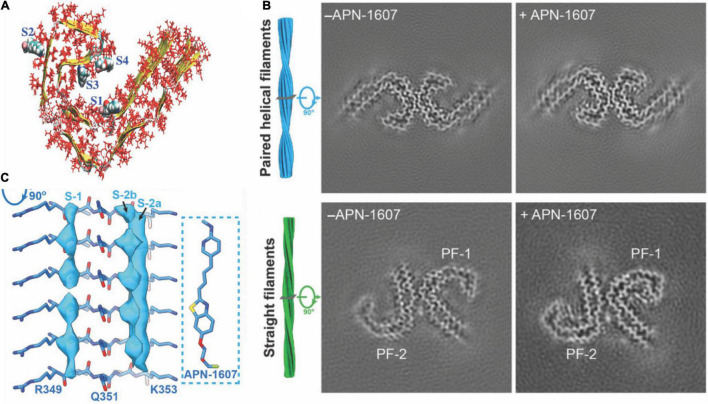
Structural and binding properties of the tau protein. **(A)** Side view of [^18^F]PM-PBB3 binding to straight filaments in AD. **(B)** Images from cryo-EM showing paired helical filaments and straight filaments in AD with bound [^18^F]PM-PBB3 (+APN-1607) and without [^18^F]PM-PBB3 (–APN-1607). Reproduced from Shi et al. (2021b) with permission from Springer Nature. **(C)** Chemical structure of a tau protofibril, representing the four high-affinity binding sites for tau tracers. S1, S3, and S4 show the core sites, and S2 shows the surface site. Reproduced from Murugan et al. (2018) with permission from American Chemical Society.

Six isoforms of microtubule-associated protein tau exist, differing in the number of repeating domains. Each isoform has either three repeats (3R) or four repeats (4R) located at the C-terminus (Spillantini and Goedert, 2013). While 4R tau is abundant in individuals with CBD and PSP, both forms are present in AD and 3R in Pick’s disease (PiD) (Spillantini and Goedert, 2013; Shi et al., 2021b). The highly conserved repeating regions are where tau proteins bind to the microtubule inside the neuron, assuring its stability. The tau protein, assisting in stabilizing microtubules, generally stays in an unfolded and phosphorylated state. When the tau protein changes to a hyperphosphorylated conformation, the involvement with microtubules decreases, and the protein migrates and congregates in the form of protofibrils (Figure 1A), the so-called paired helical filaments (PHFs) (Spillantini and Goedert, 2013).

### First-Generation Tau Tracers

The first-generation tau PET tracers include [^18^F]FDDNP, [^18^F]AV1451 (also called [^18^F]flortaucipir), and [^11^C]PBB3, and the 2-arylquinolines derivative tracers include [^18^F]THK523, [^18^F]THK5105, [^18^F]THK5117 [^18^F]THK5317, and [^18^F]THK5351 (Okamura et al., 2013).

#### *In silico* and *in vitro*

[^18^F]FDDNP, the first PET biomarker labeling NFTs, can recognize Aβ and NFTs in the brains of living humans (Agdeppa et al., 2001). There is also evidence for the retention of FDDNP in the brains from patients with other diseases, such as Down’s syndrome, prion disease and PSP. Findings obtained from *in vivo* imaging-autopsy comparison suggest a correlation between *in vivo* cortical [^18^F]FDDNP binding and the quantity of Aβ and tau build-ups at postmortem (Agdeppa et al., 2001). [^18^F]flortaucipir, is the first PET tau tracer that was recently approved to detect tau inclusions in AD by the FDA (Barthel, 2020). *In vitro* autoradiography demonstrated the binding of the radioligand [^11^C]PBB3 to the neocortex of human brains with AD compared to controls (Ono et al., 2017). Autoradiographic data from [^18^F]THK5105 indicated selective recognition of NFTs and neuropil threads in the hippocampus of AD patients (Lemoine et al., 2017). Nonetheless, off-target binding of [^18^F]THK5351 to monoamine oxidase B (MAO-B) in the basal ganglia and cortex remains a major drawback when interpreting PET images (Harada et al., 2016; Ng et al., 2017; Nihashi et al., 2021). First-generation tau tracers [^18^F]THK5351, [^11^C]PBB3 and [^18^F] flortaucipir all bind to the 3R + 4R combination of tau as in AD (Table 1; Chien et al., 2013; Maruyama et al., 2013; Okamura et al., 2013; Xia et al., 2013; Harada et al., 2016; Betthauser et al., 2017; Ono et al., 2017). Four binding sites for radioligands have been identified on the tau fibrils, where [^11^C]THK5351, [^11^C]PBB3, and [^18^F]flortaucipir bind preferentially to one or more binding sites (Murugan et al., 2018, 2021; Table 1).

**TABLE 1:** Binding properties of tau PET tracers.

Tracers	Affinity [nM]	Binding to AD (3R + 4R)/ PSP (4R)/ CBD (4R)	*In silico* binding site (S1 – S4)	References
**1st generation**				
[^18^F]THK5317		AD		Okamura et al., 2013
[^11^C]THK5351	2.9 (AD)	AD PSP CBD	1–4 Most strongly S1 and S3	Harada et al., 2016; Betthauser et al., 2017
[^11^C]PBB3	2.5, 6.3 (AD) 4.8 (PSP)	AD PSP CBD	1–4 Most strongly S4	Maruyama et al., 2013; Ono et al., 2017
[^18^F]AV1451	14.6, 0.7	AD	1–4 Most strongly S1	Chien et al., 2013; Xia et al., 2013
**2nd generation**				
[^18^F]RO948	18.5	AD	1–4	Honer et al., 2018; Yap et al., 2021
[^18^F]MK6240	0.32, 0.15, 0.3	AD	1–4 Most strongly S1	Walji et al., 2016; Malarte et al., 2021; Yap et al., 2021
[^18^F]PI-2620	4.9, 1.8 (AD)	AD PSP CBD		Mueller et al., 2017; Kroth et al., 2021; Tezuka et al., 2021
[^18^F]JNJ311	8	AD	1–4	Declercq et al., 2017
[^18^F]PM-PBB3	7.63 (AD) 3.44 (PSP)	AD PSP CBD		Tagai et al., 2021
[^18^F]GTP1	14.9	AD		Lois et al., 2019; Teng et al., 2019

#### In vivo

**FIGURE 2:**
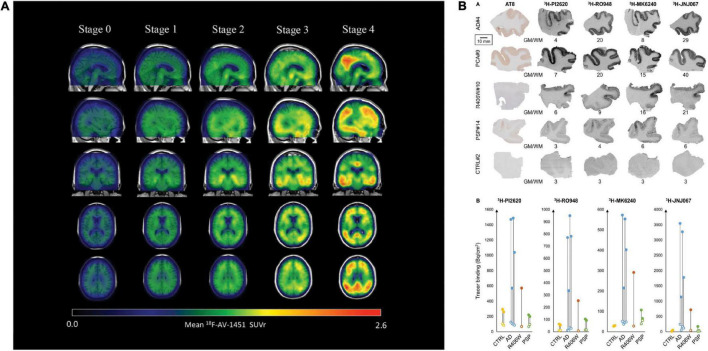
Disease staging with the tau PET tracer [^18^F]AV1451 and autoradiography with [^3^H]PI-2620, [^3^H]MK6240, [^3^H]RO948, and [^3^H]JNJ067. **(A)** Representation of tau PET images labeled with flortaucipir ([^18^F]AV1451) for staging tau pathology. An increase in SUVR is visible in the cortex and subcortex from stage 0 to 4. Stage 0 represents tau levels of healthy controls. In stage 1, tau levels are elevated in medial temporal areas. [^18^F]AV1451 accumulates in extramedial temporal regions in stage 2, followed by higher SUVRs in the inferior and lateral temporal lobes in stage 3. Significantly increased uptake finally occurred in the neocortex in stage 4. Reproduced from Chen et al. (2021) with permission from Springer Nature. **(B)** Characteristics of the binding properties of the tau tracers [^3^H]PI-2620, [^3^H]MK6240, [^3^H]RO948, and [^3^H]JNJ067 in the medial temporal lobe of patients with AD, primary tauopathies and healthy controls. (*R406W* = FTD), here a *MAPT R406W* missense mutation leads to the formation of NTFs in the case of FTD with parkinsonism linked to chromosome 17 (FTDP-17), PCA, posterior cortical atrophy; PSP, progressive supranuclear palsy. Reproduced from Yap et al. (2021) with permission from Oxford press.

[^18^F]flortaucipir showed rapid clearance from the plasma and favorable binding kinetics and has been widely used in imaging tau distribution in patients with AD (Schöll et al., 2016). [^18^F]flortaucipir enabled monitoring of tau spread and disease progression in AD (example in Figure 2A; Vogel et al., 2020; Chen et al., 2021); however, it does not detect primary tauopathies such as PSP and CBD (Fleisher et al., 2020; Malpetti et al., 2021; Van Wambeke et al., 2021). Using [^18^F]flortaucipir PET, Vogel et al. (2020) showed that tau pathology was accompanied by neuronal loss and consequent shrinkage of the brain, especially in the cortex and hippocampus of patients with AD. Meisl et al. (2021) further showed using [^18^F]flortaucipir PET and modeling that tauopathies either progress by spreading or replicating the proteopathic seeds. Another [^18^F]flortaucipir PET study by Yushkevich et al. (2021) showed that the spread of tau originated in the transentorhinal region of the medial temporal lobe, the locus coeruleus and the dorsal raphe nucleus, progress to limbic and isocortical regions in patient with AD. Vogel et al. (2021) revealed four different spatiotemporal pathways for tau pathology using [^18^F]flortaucipir PET: tau spread via posterior and lateral temporal patterns in atypical forms of AD. One limitation of [^18^F]flortaucipir is that the ligand binds non-specifically to MAO-B in the thalamus and the basal ganglia to a certain extent (Murugan et al., 2019; Wolters et al., 2021). When comparing [^18^F]THK5317 to [^18^F]THK5351, the latter was cleared faster from the gray matter in areas of the brain typically affected by tau inclusions (Betthauser et al., 2017), thus exhibiting more favorable pharmacokinetics than [^18^F]THK5317. Chiotis et al. showed that [^18^F]THK5317 accurately predicts the degree of cognitive decline in patients with AD (Chiotis et al., 2020). Nihashi et al. (2021) suggested that the quantity of [^18^F]THK5351 uptake in the brain of AD patients inversely correlated with hippocampal volume and neuropsychological assessment, implying that [^18^F]THK5351 may be a candidate for monitoring AD disease progression. Ezura et al. (2021) compared differential binding of [^18^F]THK5351 to different tauopathies and found that high [^18^F]THK5351 uptake was detected in the precentral and postcentral gyri of CBD patients, in the midbrain of PSP patients and in the parahippocampal gyri of AD patients compared to healthy controls, clearly distinguishing different tauopathies. *In vivo* imaging using [^11^C]PBB3 showed the detection of tau inclusions in the brain stem of PS19 and cortical and hippocampal regions of the rTg4510 mouse model of FTD (Maruyama et al., 2013; Ishikawa et al., 2018; Ni et al., 2018; Vagenknecht et al., 2022). A disadvantage of radiolabeled [^11^C]PBB3 is the insufficient dynamic range, clearance rate and off-target binding in the basal ganglia (Okamura et al., 2018).

### Second-Generation Tau Tracers

Several second-generation tau PET tracers with improved binding properties have been developed, including [^18^F]MK6240, [^18^F]PM-PBB3, [^18^F]RO948, [^18^F]PI-2620, [^18^F]JNJ311, and [^18^F]Genentech Tau Probe 1 (GTP1) (Leuzy et al., 2019).

#### *In silico* and *in vitro*

Hostetler et al. showed that [^18^F]MK6240 displayed selective high-affinity binding to NFT and almost no binding to Aβ. *In vitro* results comparing AD and control brains of the entorhinal cortex and hippocampus also showed high selectivity for NTF-associated regions (Hostetler et al., 2016). Tagai et al. reported that [^18^F]PM-PBB3 ([^18^F]APN-1607) overcame the limitation of the first-generation tracer [^11^C]PBB3 and demonstrated better binding properties. A cryo-electron microscopy (cryo-EM) study further showed that the different morphologies of [^18^F]PM-PBB3 were associated with paired helical filaments and straight filaments in AD (Figures 1B,C; Shi et al., 2021a). [^3^H]PI-2620 has also been shown to bind to 4R tau, thus allowing the assessment of patients with PSP (Figure 2B; Kroth et al., 2019; Brendel et al., 2020; Song et al., 2021). Additionally, [^18^F]PI-2620 has shown lower binding affinity to monoamine oxidase A (MAO-A) of AD brain homogenates (Kroth et al., 2021). Zhou et al. (2021) investigated the binding properties of PET tracers [^18^F]PI-2620, [^18^F]CBD2115, [^18^F]PM-PBB3, and [^18^F]MK6240 binding to CBD tau by using *in silico* studies where high affinity binding for the core and an entry site of the 4R tau fibril were detected. For AD tau, [^18^F]CBD2115 and [^18^F]PM-PBB3 displayed higher affinity to tau in the brain from patients with AD than [^18^F]PI-2620. However, none of the four tracers bound preferentially to 4R tau over a combination of tau species, indicating that they did not exclusively bind to 4R tau (Zhou et al., 2021). [^18^F]JNJ311, synthesized from the trimethylammonium precursor, showed promising pharmacokinetic results based on preclinical imaging: sufficient uptake in the mouse brain, followed by a rapid wash-out were detected (Declercq et al., 2017). While autoradiography study showed that [^18^F]JNJ311 detected tau inclusions in AD brain slices, it did not bind to the tau inclusions in the postmortem brain from PSP or CBD (Declercq et al., 2017). Furthermore, *in silico* findings concluded that [^18^F]JNJ311 binds to all four binding sites on the tau fibril but most strongly to sites one and two (Murugan et al., 2018). [^18^F]GTP1 showed higher binding to tau than Aβ plaques in the cortex and almost no affinity for MAO-B in brain tissue from patients with AD (Sanabria Bohórquez et al., 2019).

#### In vivo

[^18^F]RO948 has shown higher specificity for AD-related tau than other tauopathies, such as Pick’s disease, suggesting that it is an ideal biomarker for the differential diagnosis of AD (Leuzy et al., 2020). [^18^F]RO948 imaging in patients with mild AD revealed little off-target binding and outstanding kinetic properties (Kuwabara et al., 2018). Kuwabara et al. (2018) further introduced an index, tau-positive fraction (TPF), for indicating the amounts of tau in medial and lateral temporal lobe and parietal lobe with binary readout (positive or negative). Janelidze et al. (2021) recently showed that the plasma p-Tau217 levels correlated with tau accumulation measured by using [^18^F]RO948 PET in patients with early AD. The evaluation of *in vivo* tau load based on SUVRs presented evidence that [^18^F]MK6240 is a favorable biomarker to assess the presence and amount of NFT in the hippocampus of AD brains (Pascoal et al., 2018). However, Smith et al. (2021) reported sex differences in off-target binding in the meninges: cognitively unimpaired females showed more pronounced off-target binding than males for all three tracers: [^18^F]flortaucipir, [^18^F]RO948 and [^18^F]MK6240. Levy et al. (2021) demonstrated that [^18^F]MK6240 showed significant binding in the temporal lobes and the basal ganglia in Aβ-negative patients with*P301L* and *R406W MAPT* mutations and minimal off-target binding, suggesting the potential of this tracer for differential diagnosis. Sufficient [^18^F]PM-PBB3 uptake in the cortex and the hippocampus of rTg4510 mice was observed (Weng et al., 2020; Tagai et al., 2021). In patients with CBD and PSP, *in vivo* PET scans revealed elevated [^18^F]PM-PBB3 binding in the subcortex. Mueller et al. (2020) reported that [^18^F]PI-2620 showed significantly higher SUVRs and distribution value ratios (DVRs) using visual and quantitative assessments in the cortex of AD patients than in controls. In contrast, a study investigating the potential 4R imaging agent [^18^F]PI-2620 for a variety of tauopathies, such as PSP, CBD, and CBS (corticobasal syndrome), versus healthy controls indicated increased tracer uptake in the globus pallidus in patients with PSP, CBD, and CBS but also in healthy controls (Tezuka et al., 2021). Favorable imaging results have been reported for another 2nd generation tau tracer [^18^F]GTP1: Teng et al. (2019, 2021) demonstrated the association between increased tau load ([^18^F]GTP1 uptake) and cognitive decline in people with mild and progressive AD. In addition, [^18^F]GTP1 brain uptake (SUVR) has been shown negatively correlated with CSF ratio of tau368 and t-tau in patients with AD (Blennow et al., 2020).

## Discussion

To date, there have been several approved Aβ tracers, and emerging tau PET tracers with improved specificity and binding properties for the detection of NFTs in AD. Amyloid and tau PET helps to uncover the interplay between Aβ, tau and neurodegeneration in longitudinal studies of the disease progression. Aβ PET has been established as diagnostic tool for assisting clinical diagnosis, while the diagnostic value for tau imaging has yet to be further demonstrated (Altomare et al., 2021). Tau PET has a strong impact on diagnosis and on subsequent cognitive decline in AD (Ossenkoppele et al., 2021). Recent head-to-head comparison study (tau PET vs. amyloid PE vs. MRI) has demonstrated the accuracy and added prognostic value of tau PET in patients with preclinical and prodromal AD (Ossenkoppele et al., 2021). Finding an optimal imaging biomarker remains a demanding task, as there are several prerequisite for PET tracers targeting at central nervous system, including structural requirements such as the size to pass the BBB, pharmacokinetic properties, and stability of the chemical for imaging. Off-target binding of tau radiotracers, e.g., to MAO-B, is a concern for first-generation tau PET tracers (Murugan et al., 2019). Other off-target binding sites include neuromelanin and melanin-containing cells (Aguero et al., 2019), which [^18^F]MK6240 tends to bind. The novel tracer [^18^F]SNFT-1 (THK5562) might have overcome this drawback, as it demonstrated reduced off-target binding to MAO enzymes in preclinical animal experiments (Ishiki et al., 2020).

While eminent research has been conducted on Aβ imaging, more insight into how Aβ structures, such as fibrils or protofibrils, are associated with pathology are to be provided. Having the same amino acid sequence of Aβ, aged non-human primates develop Aβ deposition similar to humans, raising the question of why they lack the human pathological manifestation and that resulted in the hypothesis of conformational changes on a molecular level (Rosen et al., 2016). Absence of [^11^C]PiB binding to Aβ aggregates in the brains from monkeys with age-related amyloid plaques was reported (Rosen et al., 2011, 2016). Studies have further shown that binding is dependent on plaque structure (dense or diffuse) or on the relative ratios of Aβ_40_ and Aβ_42_ (Aβ_42_/Aβ_40_) (Lee et al., 2019). Recent cryo-EM study found that the structure of Aβ fibrils from human AD patients were different from those formed *in vitro* (Kollmer et al., 2019). Contrary to synthetic fibrils that form left-hand twists, the human-derived fibrils demonstrated right-handed twists. Nonetheless, the folding of proteins and the assembly of protofilaments are conserved structures. Autosomal dominant AD is the genetic variant of AD and emerges due to mutations in the presenilin 1 (*PSEN1*), presenilin 2 (*PSEN2*), and amyloid precursor protein (*APP*) genes (Gordon et al., 2018). Yang et al. (2022) reported that different structures were observed in fibrillar Aβ from sporadic and autosomal dominant AD. Lower Aβ detection by [^11^C]PiB might be associated with morphological changes of Aβ molecules in genetic forms of AD or other species expressing amyloidosis (Schöll et al., 2012). Longitudinal observations of subjects with the *PSEN1 E280A* mutation without clinical symptoms served as a tool to monitor the association between Aβ, entorhinal tau accumulation and cognitive decline (Sanchez et al., 2021). Studies found only minimal [^11^C]PiB retention in the cortex of APP arctic mutation carriers versus non-carriers while the CSF Aβ_42_ and p-tau are pathologic, thus implying the importance of genetic variants on Aβ structure (Lemoine et al., 2021). In contrast, Benzinger et al. (2013) discovered that the [^11^C]PiB cortical retention was elevated in autosomal dominant AD even 15 years before the onset of the disease.

## Conclusion

Positron emission tomography imaging using Aβ and tau tracers has enabled the early and differential diagnosis of AD and monitoring of disease progression. Although many Aβ PET tracers has been applied in preclinical research and a few for clinical use, there is only one tau PET imaging biomarker available in the clinic. For the differential diagnosis of primary tauopathies, such as CBD and PSP, there is no specific 4R tau PET tracer to enable specific detection, which requires future development. In addition, the detection of other targets, neuroinflammation and synaptic density at the beginning of AD has the potential for more holistic *in vivo* diagnostics.

## Author Contributions

CM and RN wrote the draft manuscript. Both authors contributed to the manuscript.

## Conflict of Interest

The authors declare that the research was conducted in the absence of any commercial or financial relationships that could be construed as a potential conflict of interest.

## Publisher’s Note

All claims expressed in this article are solely those of the authors and do not necessarily represent those of their affiliated organizations, or those of the publisher, the editors and the reviewers. Any product that may be evaluated in this article, or claim that may be made by its manufacturer, is not guaranteed or endorsed by the publisher.
